# Incisional Hernia Development: Wound Healing Gone Wrong?

**DOI:** 10.1111/wrr.70131

**Published:** 2026-03-05

**Authors:** Asim A. Abbas, Paul Gunning, Srinivas Chintapatla, Roland Kröger

**Affiliations:** ^1^ York Abdominal Wall Unit, Department of General Surgery York and Scarborough Teaching Hospitals NHSFT York UK; ^2^ Department of Biology University of York York UK; ^3^ School of Physics, Engineering and Technology University of York York UK

## Abstract

Incisional hernia (IH) is a common complication of abdominal surgeries, characterised by the protrusion of abdominal contents through a weakened surgical scar. Despite advancements in surgical techniques and biomaterials, IH remains a significant clinical and economic burden, with recurrence rates reaching up to 32% after repair. This unusually high number, despite many years of research focused on the improvement of surgical techniques, requires a better understanding of the potential origins of IH occurrence. This must implicate the tissues involved in scar formation of the abdominal wall for a better understanding of how these tissues are affected by the incision and what can potentially affect the most optimal wound healing. This work aimed to provide a comprehensive summary of the current knowledge regarding the ultrastructure of the abdominal wall and how incisions affect its mechanical integrity.

## 1 Introduction

Incisional hernia (IH) is a common and challenging postoperative complication that occurs when the integrity of the abdominal wall is compromised at the site of a surgical incision, leading to the formation of a bulge, detected either by clinical examination or imaging [1]. Characterised by the protrusion of abdominal contents through a weakened scar, IH not only presents a significant burden for patients but also imposes substantial healthcare costs globally [2, 3]. Reported incidence rates vary widely across the literature, ranging from approximately 5% to 20% following abdominal surgeries [4], depending on factors such as surgical technique, patient comorbidities and follow‐up duration [5]. In complex cases, the incidence can rise, particularly in patients with obesity, diabetes, or previous hernia repairs. The clinical impact of IH extends beyond the need for secondary surgical intervention. Patients with IH often experience chronic pain, reduced quality of life, negative psychological consequences and embarrassment, and limitations in physical activity [6, 7]. Complications such as bowel obstruction, incarceration, or strangulation can lead to life‐threatening emergencies [8]. As the prevalence of abdominal surgeries increases, preventing IH has become a pressing priority for both surgeons and researchers. IH development is primarily attributed to failure in the wound healing process [9]. While traditional views have focused on surgical and patient‐related risk factors, emerging evidence suggests that IH may be driven by underlying dysfunctions in the biological and mechanical pathways of wound repair. Disruptions in collagen metabolism, matrix remodelling and biomechanical signalling during post‐laparotomy healing appear to have a role in scar fragility and hernia recurrence. Despite advancements in surgical techniques and biomaterials, long‐term outcomes for IH repairs remain poor, with recurrence rates as high as 32% even after mesh implantation [10]. There is a growing need to explore the pathophysiological mechanisms underlying IH, shifting focus from purely technical considerations to the molecular, cellular and biomechanical factors that govern abdominal wall integrity. This review proposes that IH arises from dysfunctional wound healing processes and seeks to explore the interplay of collagen alterations, scar remodelling and pathological mechanisms such as dystrophic calcification (DC) (mineralisation). By examining the molecular and biomechanical pathways involved, we aim to highlight gaps in current knowledge, which is crucial to identify potential avenues for improving IH prevention and treatment.

## 2 The Ultrastructure of the Abdominal Wall

**FIGURE 1:**
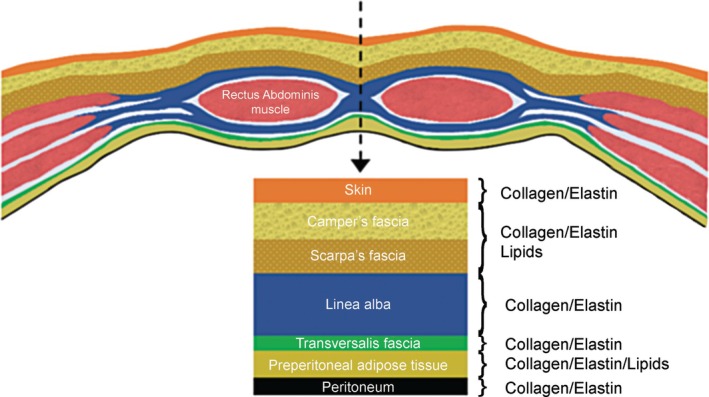
Tissue layers involved in the abdomen with intercalated collagen/elastin and lipid‐based tissues.

The tissue organisation in the abdominal wall consists of a complex combination of collagenous and lipid layers as shown in Figure 1. Operations on the abdomen require an incision through this multilayer of tissues, which plays a key role in the healing process. As each layer consists of a characteristic tissue type, their ultrastructure, composition and interfaces with each other need to be understood when dealing with issues related to abdominal wall reconstruction and possible origins of hernia formation.

### 2.1 Skin

The skin forms the outermost barrier of the abdominal wall and comprises two primary layers: the epidermis and dermis. The epidermis consists primarily of keratinocytes, providing a protective outer layer, while the dermis houses blood vessels, nerve endings and a fibrous network of collagen and elastin that offers strength and elasticity [11, 12]. Collagen fibrils in the dermis are arranged in a basket‐weave formation, a structural organisation that enables multidirectional resistance to mechanical forces [13, 14]. This arrangement supports the skin's integrity under varying conditions, such as stretching and compression during abdominal wall motion. Lines of equal tension within the dermis, known as Langer's lines, reflect the predominant alignment of collagen fibres [15]. On the abdomen, these lines run transversely with slight curvature. Surgical incisions parallel to Langer's lines heal more efficiently, with fewer complications and improved cosmetic outcomes. In contrast, longitudinal incisions, like midline laparotomies, disrupt the natural alignment of collagen fibres, leading to increased wound tension, broader scars and a higher risk of complications.

### 2.2 Subcutaneous Tissue

The subcutaneous layer, situated beneath the dermis, consists of two distinct fasciae: Camper's fascia and Scarpa's fascia. *Camper's fascia* is a superficial layer rich in fatty lobules embedded within a fibrous meshwork. It acts as a shock absorber, mitigating mechanical impact, and provides a cushioning effect and thermal insulation to the abdominal wall. In healthy individuals, this layer supports the structural integrity of the abdominal wall, limiting the vertical extension of hernias [16]. However, in obesity, excess adipose tissue accumulates in Camper's fascia, altering its mechanical properties. This predisposes patients to IH formation, as obesity is a well‐documented risk factor for herniation. *Scarpa's fascia* is a deeper, dense fibrous layer composed of collagen and elastin, with fibres aligned to withstand unidirectional forces. The principal role of Scarpa's fascia is thought to be to anchor the superficial layers to the underlying linea alba, supporting the midline abdominal integrity during dynamic loading conditions [17]. Scarpa's fascia may also prevent sagging and minimise postoperative fluid accumulation. Scarring or fibrosis within Scarpa's fascia, whether due to injury or surgery, can stiffen this layer, ultimately compromising the structural support provided to the linea alba and increasing the risk of herniation [18].

### 2.3 Linea Alba

The linea alba is a midline tendinous structure in the anterior abdominal wall, formed by the interweaving aponeuroses of the abdominal muscles. Functionally, it resembles tendons or ligaments rather than fascia, providing primary tensile strength to the abdominal wall and counteracting constant intra‐abdominal pressure [19]. The linea alba endures significantly greater forces than the overlying skin and subcutaneous layers, making its closure during midline laparotomies crucial for preventing IH formation. Its densely packed collagen matrix delivers strength and elasticity, with a smaller proportion of elastin fibres aligned to mirror collagen fibre orientations [15]. This alignment ensures elastic recoil and effective tension distribution. In IH patients, alterations in collagen type I/III ratios have been observed, reducing the tensile strength of the linea alba and predisposing it to failure under stress [20]. The collagen arrangement in the linea alba creates an anisotropic network [21, 22] exhibiting different mechanical properties depending on the direction of applied stress. Three distinct layers of collagen fibres are distinguished: a layer of (1) intermingling oblique fibres that contribute to multidirectional resistance, (2) a regular transverse fibre layer that imparts transverse stiffness and (3) inconstant, small irregular oblique fibres, which add variability to its structure [23]. Gräbel et al. [22] used biomechanical measurements to show that the linea alba extends the most in the longitudinal direction and the least in the transverse direction, a property attributed to its high density of transverse collagen fibres. This transverse resistance is key in counteracting the constant intra‐abdominal pressure generated during activities such as coughing, lifting or straining. Any weakening of this resistance due to collagen imbalance or surgical compromise significantly increases the risk of hernia formation.

**FIGURE 2:**
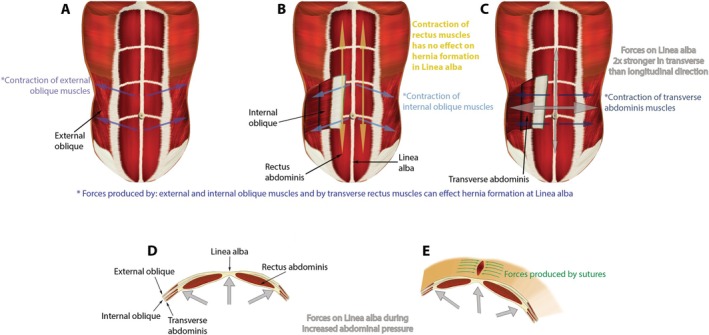
Biomechanical forces acting on the linea alba during physiological loading and post‐suture closure. Panels (A–C) demonstrate the directional forces exerted by the external oblique, internal oblique and transverse abdominis muscles. Notably, rectus abdominis contraction does not contribute to midline tension (Panel B). Panel (D) illustrates how increased intra‐abdominal pressure amplifies transverse strain on the linea alba, while. Panel (E) shows the opposing forces generated by sutures post‐laparotomy.

These biomechanical relationships are illustrated in Figure 2, which demonstrates the directional forces applied to the linea alba by the external oblique, internal oblique and transverse abdominis muscles. As shown, transverse forces acting on the midline substantially exceed longitudinal forces, and rectus abdominis contraction contributes minimally to linea alba tension. The figure further highlights how increased intra‐abdominal pressure and the counteracting forces generated by sutures create opposing stress vectors across the midline. This visualisation reinforces the anisotropic load‐bearing behaviour of the linea alba and demonstrates why midline closure techniques must respect its direction‐dependent mechanical properties. Panels D and E of the figure highlight how intra‐abdominal pressure and suture forces produce opposing mechanical stresses, reinforcing the need for a closure strategy that respects the anisotropic tension‐bearing properties of the abdominal wall.

The fibre alignment also corresponds to the Langer's lines in the skin. Langer's lines are topographical lines on the skin that correspond to the predominant orientation of collagen fibres in the dermis, guiding the natural direction of skin tension. Under loading conditions, the oblique fibres align transversely, enhancing structural stiffness and resistance to intra‐abdominal pressure. Additionally, the role of the linea alba in distributing mechanical stress across the abdominal wall is facilitated by its interaction with adjacent layers such as the rectus sheath and transversalis fascia [24]. These layers function synergistically to maintain abdominal wall integrity. Disruption of this balance, such as through scarring or poor surgical technique, can precipitate localised weaknesses that predispose to hernia formation. Failure to achieve proper tension or alignment during surgical closure of the linea alba can lead to hernia recurrence. Mesh manufacturers have attempted to replicate the linea alba's anisotropic properties in repair materials; however, the exact mechanisms of its healing and long‐term repair remain poorly understood [25].

### 2.4 Transversalis Fascia, Preperitoneal Adipose Tissue and the Peritoneum

The transversalis fascia is a weak, fibrous layer covering the deeper surface of the transversus abdominis muscles. It is separated from the peritoneum by a layer of adipose tissue, known as preperitoneal fat [26]. While it is relatively weak compared with the linea alba or rectus sheath, it contributes to abdominal wall stability. Situated between the transversalis fascia and the peritoneum, the preperitoneal adipose tissue acts as a cushion, protecting abdominal structures from direct mechanical impact. This layer, often prominent in individuals with higher body fat, can exacerbate hernia risk due to increased intra‐abdominal pressure and decreased tensile resistance of the abdominal wall. The peritoneum is a serous membrane primarily composed of collagen and elastin fibres, conferring both strength and flexibility [27, 28]. It may play an indirect role in hernia formation as it forms the hernia sac. Although some surgical techniques involve closing the peritoneum to enhance repair strength, studies indicate that this step does not contribute significantly to overall abdominal wall stability and it is unclear if this changes the risk of adhesion formation [29, 30].

### 2.5 Biomechanical Perspective

The abdominal cavity operates as a dynamic biomechanical unit, enclosed by muscles, tendinous membranes and skeletal structures [31]. It is a three‐dimensional ovoid cavity with the diaphragm forming the domed roof and the pelvic floor forming the narrow, inverted bowl. The relationship between intra‐abdominal pressure (up to ~0.02 MPa) and the mechanical properties of the abdominal wall ensures effective load distribution during various physical activities, including coughing, defecation, or lifting [23]. Hernias typically develop when the structural integrity of the abdominal wall layers is compromised by factors such as increased intra‐abdominal pressure, obesity, or poor surgical technique. Laplace's law explains the mechanism of extension, as tension becomes concentrated at the hernia defect, worsening the protrusion over time. Effective surgical repair must consider restoring the balance of forces across the abdominal wall. Normal intra‐abdominal pressure exerts forces on the linea alba estimated at ~4500 N/m transversely and ~1500 N/m longitudinally [23]. When structural tissues are unable to contain this pressure, the abdominal wall ruptures at its weakest point, leading to hernia formation. This process aligns with Laplace's law, which predicts that wall tension increases at regions with the largest radius and thinnest wall.

**Challenge:**

As it increasingly emerges that the microstructural and sub‐microstructural organisation of collagen is pivotal for the macroscopic physical, particularly mechanical properties of connective tissue, the following key questions need to be addressed to identify potential detrimental factors affecting the structure of the abdominal wall:

- How do changes in the microscopic structure of the collagen–elastin network within the linea alba contribute specifically to hernia susceptibility at different stages of life or disease?

Collagen and elastin form a tightly regulated structural network crucial for the mechanical integrity of the linea alba. However, the microstructural organisation and composition of these fibres can vary significantly with aging, chronic disease, or genetic predispositions. Despite recognising a correlation between collagen I/III ratios and hernia susceptibility, the exact morphological changes, such as alterations in fibril diameter, cross‐link density, or elastin integrity, have not yet been fully characterised at various life stages. Clarifying these structural nuances could provide considerations into why certain populations, such as elderly patients or those with connective tissue disorders, exhibit greater hernia risks. This understanding would help in developing patient‐specific preventive and therapeutic strategies, potentially targeting specific molecular or mechanical vulnerabilities in the linea alba.

B Which exact mechanical and biochemical signals determine optimal healing and remodelling of the linea alba post‐surgery?

While the mechanical and biochemical environments clearly influence tissue healing, precise knowledge of their interactions in abdominal wound repair remains limited. Although fibroblast activity, extracellular matrix deposition and remodelling are known to be mechanically and biochemically modulated, the exact signalling pathways governing these processes—particularly in abdominal fascia—are not well elucidated. Determining these signals is crucial to optimise surgical closure techniques and postoperative care protocols. A comprehensive understanding could inform bioengineering of synthetic or biological meshes and the development of pharmaceuticals or growth‐factor therapies that facilitate optimal collagen remodelling and accelerate recovery post‐surgery, thereby reducing the incidence of IHs.

C Can targeted modifications in surgical techniques or materials effectively mimic the natural anisotropic properties of the linea alba to reduce hernia incidence?

Current surgical meshes and closure techniques aim to provide strength and support to the abdominal wall postoperatively; however, they rarely replicate the precise anisotropic (directionally dependent) mechanical properties intrinsic to the linea alba. Such anisotropy is essential in providing effective resistance to forces generated by intra‐abdominal pressures from activities like coughing, bending and lifting. Investigating whether modern bioengineering approaches could produce meshes with mechanical properties tailored specifically to mimic the natural anisotropy of the linea alba could be transformative. Addressing this question could significantly reduce complications and recurrence rates, especially in high‐risk patients undergoing complex abdominal wall reconstruction (CAWR) surgery.

D How do obesity‐related changes in the preperitoneal adipose tissue mechanistically interact with collagen remodelling and contribute to IH formation?

Obesity is a well‐established risk factor for IH formation, yet the specific mechanisms linking obesity to increased hernia susceptibility remain incompletely understood. Increased adipose tissue accumulation within the preperitoneal layer not only mechanically weakens the abdominal wall by exerting continuous tension but may also biochemically impair collagen synthesis, remodelling and cross‐linking through chronic low‐grade inflammation and altered adipokine signalling. Understanding these mechanistic pathways at a cellular and molecular level would develop a fuller understanding of how adipose‐derived factors influence fibroblast function, inflammation and collagen turnover. This could enable targeted therapeutic interventions in obese populations.

## 3 Tissue Remodelling and Wound Healing in IH Development

**FIGURE 3:**
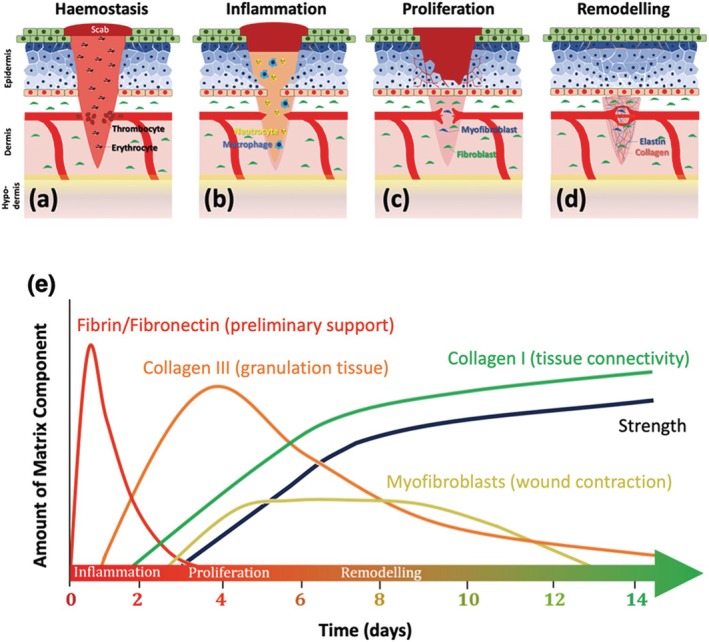
Time evolution of tissue formation during wound healing. After initial inflammation and fibrin/fibronectin proliferation, collagen III is produced for rapid closure before collagen I and fibroblasts are gradually delivered for wound contraction and increasing wound breaking strength. Sequence of skin wound healing process. Homeostasis (a) is followed by inflammation (b), proliferation (c) and remodelling (e). The expression of tissue repair components over time during wound healing.

Most scientific publications concentrate on cutaneous wound healing in isolated and static monolayer models as shown in Figure 3. However, most incisions, like the abdominal midline, affect a multitude of layers within a dynamic environment, which becomes more challenging to model [32].

**FIGURE 4:**
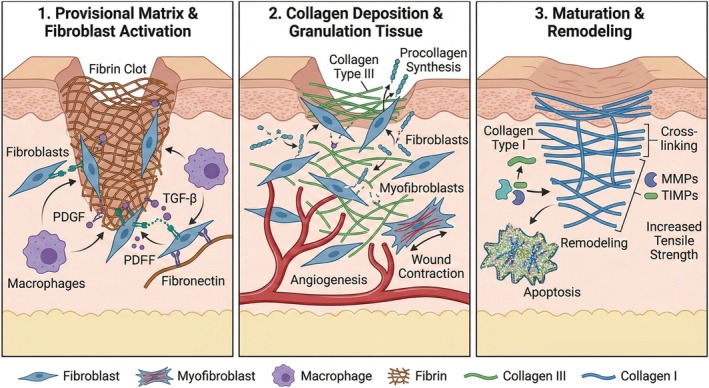
Phases of wound healing with associated collagen deposition and remodelling.

Animal models have provided us with some understanding on the molecular and mechanical particularities of incisional wound healing, but research in human subjects remains limited, often focusing on skin grafts and burn wounds [32, 33]. The complexity of abdominal incisions requires a deeper investigation into the interplay between multiple tissue layers and dynamic biomechanical forces. In the immediate aftermath of a laparotomy, haemostasis occurs to control blood loss. During this phase, a fibrin–fibronectin mesh forms over the platelet plug, providing a preliminary scaffold for cellular attachment and migration. This phase, which takes place within the first few hours post‐surgery, sets the foundation for subsequent tissue repair [32, 34]. Following haemostasis, the inflammatory phase begins. Immune cells infiltrate the site to eliminate pathogens, clear damaged matrix elements and recruit fibroblasts to initiate matrix production. This phase lasts until approximately day 3 [34, 35]. The proliferative phase, commencing around day 3, is characterised by the formation of granulation tissue. This new matrix is rich in thin, parallel collagen type III fibres that provide unidirectional tensile resistance. In contrast, intact skin exhibits a basket‐weave collagen arrangement, which confers multidirectional strength. During this stage, collagen type I production begins, gradually shifting the collagen type I/III ratio to 1:2 by day 7 [36]. Granulation tissue contains double the collagen type III content compared with unwounded skin, where collagen type I dominates at 80%–85% and collagen type III constitutes 10%–15%. A proportion of fibroblasts differentiate into myofibroblasts, which uniquely express contractile stress fibres that approximate wound edges and enhance tissue closure [36, 37]. Despite the production of this new matrix, the incision has less than 5% of normal tissue strength during the first 7 days post‐laparotomy, making suture integrity critical [32, 35]. During the remodelling phase, granulation tissue is replaced by an acellular, avascular fibrous scar under the influence of local mechanical forces [38]. This phase, which is clinically significant, determines the scar's strength and appearance based on the rate, quality and extent of matrix deposition. During this time, thin, disorganised collagen type III fibres are degraded by matrix metalloproteinases (MMPs), and thick, organised bundles of collagen type I are deposited along tension lines. After 3–4 weeks, collagen synthesis and degradation reach equilibrium, and further increases in wound strength are attributed to collagen cross‐linking [39, 40] Despite these processes, maximum scar strength rarely exceeds 80% of intact tissue strength, even after prolonged remodelling [41]. The sequential interplay of cellular activity, matrix remodelling and mechanical stressors during each phase of wound healing is summarised in Figure 4, contextualising the role of collagen turnover, matrix deposition and the contribution of myofibroblasts in restoring tensile integrity.

**Challenge:**

As the processes of tissue remodelling and wound healing critically affect the resulting tissue integrity, it is important to focus on four central questions in this context:

- What specific cellular signalling pathways govern the transition from granulation tissue (type III collagen‐rich) to mature scar tissue (type I collagen‐dominant) in abdominal wounds?

The transition from early granulation tissue to mature, mechanically robust scar tissue is critical in preventing hernia formation. While it is known that early wound healing primarily involves collagen type III deposition, transitioning later to predominantly type I collagen, the precise cellular signalling pathways that regulate this transformation remain insufficiently characterised, particularly in abdominal fascia. Identifying these pathways, including growth‐factor involvement (e.g., TGF‐β), MMPs and their tissue inhibitors (TIMPs), is essential. Understanding these processes could facilitate targeted molecular therapies to enhance the speed and quality of fascial remodelling, significantly reducing the window of vulnerability during which hernias commonly develop.

B Can external modulation of mechanical forces (compression devices, controlled activity, etc.) influence collagen deposition and alignment, thereby optimising scar tissue mechanical properties?

Mechanobiology research underscores the importance of physical forces in shaping tissue architecture during wound healing. External mechanical forces applied via compression garments, controlled early postoperative physical activity, or mechanical stimulation devices might influence fibroblast orientation, collagen fibre alignment and maturation of abdominal scars. However, the precise efficacy, optimal timing and magnitude of these interventions specifically in abdominal wall healing remain unclear. Investigating these aspects would allow redefining standardised postoperative rehabilitation protocols designed to exploit mechanotransduction pathways, potentially yielding stronger and better‐aligned scar tissue and reducing hernia recurrence.

C How does obesity‐induced chronic inflammation at a molecular level specifically impact collagen remodelling and MMP/TIMP regulation during abdominal wall healing?

Obesity‐associated chronic inflammation is widely recognised as detrimental to wound healing. Inflammatory cytokines produced by hypertrophic adipocytes and adipose‐resident macrophages could profoundly alter collagen synthesis, maturation and degradation. Specifically, the dysregulated activity of MMPs and their inhibitors (TIMPs) in obese patients might lead to an imbalanced extracellular matrix turnover, yielding mechanically inferior scar tissue predisposed to herniation. Elucidating these specific molecular interactions and inflammatory signalling pathways could open new therapeutic avenues aimed at reversing or moderating inflammation‐induced impairments in abdominal wound healing.

D Can non‐invasive imaging or spectroscopic methods (such as Raman spectroscopy) accurately track real‐time collagen remodelling processes in vivo, enabling earlier intervention or risk prediction?

Currently, assessing collagen remodelling and quality postoperatively largely relies on indirect clinical indicators or invasive biopsies. Non‐invasive modalities, such as Raman spectroscopy, hold promise to provide real‐time, objective data on tissue composition, collagen alignment, maturity and cross‐linking status at various postoperative stages. Raman spectroscopy is a powerful method to analyse the molecular makeup of materials such as connective or mineralised tissue. Using a laser source, the molecules in the sample are excited and their molecular vibrations lead to a reduction of the energy of the reflected light in comparison to the primary source. The reflected light is then analysed by a spectrometer, which allows us to determine energy shifts due to the light‐sample interaction. The observed shift in energy is characteristic for the molecular structure of the sample and can be used to identify tissue composition and modifications. It can be used to distinguish different collagen types, modifications due to disorders and the presence of mineralised deposits. For highly ordered collagenous tissues, polarised Raman spectroscopy can identify variations of collagen orientation and also the presence of water in the tissue can be detected. Raman spectroscopy is generally non‐destructive and allows for a rapid tissue analysis without extensive sample preparation.

Clarifying the capability and accuracy of such imaging methods could enable early identification of abnormal healing trajectories, providing the opportunity for timely therapeutic interventions. Ultimately, these non‐invasive methods could become predictive biomarkers for IH risk, significantly enhancing personalised postoperative care and surveillance strategies.

## 4 The Hierarchical Structure of Collagen and Collagen Alterations in IH Patients

### 4.1 Collagen Hierarchy and Assembly

Collagen plays an essential role in post‐laparotomy healing and hernia development [35, 36, 37]. Connective tissues, such as fascia, owe their ability to withstand tensile and compressive forces to a fibrous scaffold predominantly composed of collagen. The specific stiffness and elasticity of tissues are determined by their collagen content and hierarchical organisation. For instance, the collagen‐rich composition and layered structure of the linea alba confer anisotropic mechanical properties, enabling the tissue to exhibit direction‐dependent stiffness and elasticity, crucial for its role in the abdominal wall [22]. Collagen assembly begins intracellularly within fibroblasts, where amino acids assemble into protocollagen strands. These strands are further twisted into a triple‐helix structure called procollagen, the basic building unit of collagen. The triple‐helix configuration is stabilised by hydroxyproline residues and secreted into the extracellular space, where end‐ligation processes convert procollagen into tropocollagen. Tropocollagen units, ~300 nm in length and < 2 nm in diameter, self‐assemble into collagen microfibrils, forming the distinctive 67 nm banding pattern due to the staggered arrangement of the tropocollagen units [13, 42, 43]. Cross‐linking between lysine and hydroxylysine residues in neighbouring molecules gives collagen its unique tensile strength [44]. This property is tissue‐specific, as fibril diameters range from 12 to 500 nm depending on the type of connective tissue. Microfibrils aggregate into higher‐order collagen bundles, which undergo further maturation processes mediated by enzymes like MMPs and their inhibitors (TIMPs). These enzymes regulate collagen cleavage, cross‐linkage and fibril maturation [45], and their imbalance is implicated in connective tissue disorders such as Ehlers‐Danlos syndrome and hernia formation. Reduced concentration of the enzyme (lysyl oxidase) which catalyses the cross‐linking process is found to be associated with certain types of hernias [34]. While many studies point towards abnormal collagen deposition in IH, the literature is not unanimous, and discrepancies in findings likely reflect variation in study design, sampling location and detection techniques.

### 4.2 Mechanical and Surgical Disruption Pathway

The formation of IHs is driven by mechanical and biological factors. Mechanical pathways involve disruptions in abdominal tension during healing, while biological pathways suggest an underlying connective tissue disorder termed ‘hernia disease’ [20, 35]. Franz stated that while primary hernias (e.g., inguinal hernia) are the result of an underlying connective tissue disorder (biological), secondary hernias like IH are induced by inappropriate tension across the abdomen [35]. Dubay and Franz however argued that optimised incisional healing depends on the normal assimilation of both biological and mechanical signals [32]. The mechanical pathway claims that most IH patients lack pre‐existing wound healing defects, with hernia development arising from inadequate mechanical signalling. Proper mechanical priming during healing is essential for fibroblasts to deposit appropriate amounts and types of collagen. However, factors such as suture tension, excess adipose tissue and abrupt intra‐abdominal pressure changes (e.g., coughing or vomiting) can impair this process. In a similar way to how bones, ligaments and musculature repair depend on active rehabilitation; surgical incisions need to be subjected to the appropriate degree of tension during each phase of healing [46]. This mechanical priming allows fibroblasts to deposit the right amount and type of collagen to heal the incision and avoid later hernia formation. Inappropriate or lack of mechanical signalling is often due to suture pulling in the metabolically active zone of healing, or poor surgical techniques (incision length, direction, suturing style). Furthermore, excess adipose tissue risk in obese patients may prevent tension free wound closure [47]. These mechanical disruptions prevent effective collagen production and deposition [34, 48, 49, 50], ultimately compromising scar integrity and predisposing to herniation.

### 4.3 Connective Tissue Disorder Pathway

The biological pathway suggests that genetic alterations underlie IH susceptibility [20]. Studies have identified mutations affecting collagen synthesis, fibril formation and cross‐linking in IH patients. These alterations disrupt the normal ratio of collagen types I and III. The resulting fibrils are thinner with reduced mechanical stability [20, 51]. This systemic defect aligns with observations of connective tissue disorders like Ehlers–Danlos syndrome. It is likely that the same or similar processes drive IH formation [52]. However, this is not clear, with some studies investigating a possible association with aortic aneurysms showing insufficient data currently available to support a systemic connective tissue defect affecting both hernia and the abdominal wall [53]. Furthermore, other studies have not confirmed these findings, suggesting that if a systemic ‘hernia signature’ exists, it may only be present in a subset of patients or may be context‐dependent.

### 4.4 Collagen Ratio Alterations and Evidence Conflicts

**FIGURE 5:**
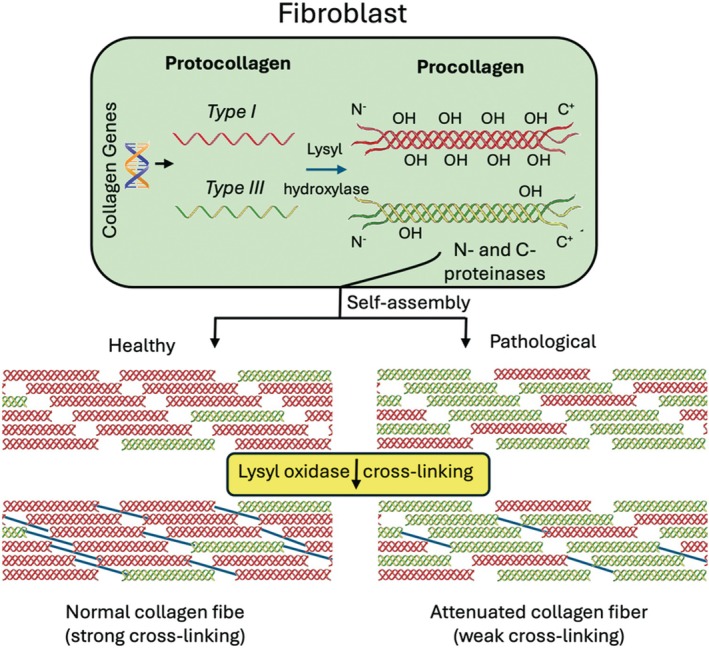
Suggested pathway for weakened (attenuated) collagen fibre from fibroblast based expression of collagen I and III resulting in lower hydroxylation of the procollagen and subsequently pathologically decreased cross‐linking of collagen III rich fibres.

Histological analyses of IH scars consistently reveal increased type III collagen, leading to a decreased type I/III ratio. Klinge et al. demonstrated elevated levels of both collagen types in hernia scars compared with intact tissues, with disproportionately higher type III levels in recurrent IH cases [54, 55] leading to the description of a ‘hernia disease’ as schematically described in Figure 5.

Rosch et al. [56, 57] investigated abdominal skin scars from patients with primary IH, recurrent IH and unrelated re‐laparotomies, finding the ratio of collagen type I/III was lower in patients with recurrent IH compared with controls. Significant changes in hernia patients compared with stable mature scars suggest that the changes are not due to normal incisional scar formation, but an abnormal finding related to IH formation [56, 57]. The generation of a mechanically inferior scar tissue, by way of deranged collagen ratios, may increase susceptibility to IH recurrence. However, not all studies demonstrate a consistent pattern. While many report increased collagen type III and reduced type I/III ratios in hernia tissue, others show considerable variability or even normal levels, likely reflecting differences in methodology, patient selection and anatomical sampling sites. Additionally, genetic studies, such as those by Henriksen et al., have highlighted systemic changes in collagen metabolism. Reduced type I/III ratios were observed in skin and fascia samples distant from hernia sites, supporting the concept of a genetic ‘hernia signature’ that predisposes individuals to abnormal wound healing [20, 58]. These findings suggest that IH development is not merely a local phenomenon but may involve systemic alterations in collagen metabolism. It is important to highlight several limitations within the current body of literature investigating collagen alterations in IH patients. However, it is important to note that not all studies have reported consistent findings regarding collagen type I/III ratios. Variability may reflect differences in patient populations, hernia classification, surgical history (e.g., open vs. laparoscopic), or technical approaches such as histochemical staining versus biochemical assays. This methodological heterogeneity presents a significant challenge in synthesising a unified mechanistic model of collagen dysregulation in IH. Moreover, there is a lack of standardisation in defining the location of IH, with some studies failing to distinguish between midline and ventral hernias. Many studies investigating systemic collagen metabolism sampled tissue away from the hernia site, whereas others focused exclusively on scar tissue. Furthermore, studies employ differing techniques, including histochemical staining, Western blotting, or biochemical quantification, each with varied sensitivity and specificity. Surgical factors, such as laparoscopic vs. open approaches, patient comorbidities and time since incision, are often inconsistently reported, further limiting direct comparison between studies. This variability makes it challenging to draw definitive conclusions about whether observed changes are local to the hernia site or indicative of systemic connective tissue alterations.

**Challenge:**

Since a deep understanding of the tissue organisation across the length‐scales is essential for the development of diagnostic and therapeutic approaches to IH prevention and treatment, the following key challenges need to be addressed:

- To what extent are the observed changes in collagen type I/III ratios in IH patients due to local mechanical disruptions versus underlying systemic genetic defects?

Current studies consistently indicate alterations in collagen metabolism in IH patients, particularly changes in the collagen type I/III ratio. However, the extent to which these alterations result from local surgical disruptions (e.g., suture tension, wound ischemia, or poor mechanical signalling) compared with underlying genetic or systemic collagen defects remains unclear. Understanding whether collagen abnormalities are intrinsic (systemic) or extrinsic (local/mechanical) has substantial implications. If primarily local factors are responsible, improving surgical techniques and postoperative mechanical management may suffice to reduce hernia incidence significantly. Conversely, if systemic genetic factors predominantly drive collagen imbalance, predictive biomarkers or targeted medical therapies may become essential to prevent hernias, particularly in genetically predisposed populations.

B How do specific genetic variations or mutations in collagen‐related genes (such as COL1A1, COL3A1, lysyl oxidase or enzymes regulating collagen cross‐linking) correlate with hernia susceptibility, severity and recurrence rates?

Although research has suggested a genetic basis (‘hernia disease’) underlying collagen abnormalities in IH patients, definitive genetic markers associated with increased hernia risk remain elusive. Detailed exploration of collagen‐related genes—particularly those coding for type I and III collagen and their regulatory enzymes—is essential. Pinpointing specific mutations or polymorphisms associated with increased susceptibility or recurrence would enable genetic screening and personalised treatment strategies, including preventive surgical measures or targeted therapeutic interventions that enhance collagen cross‐linking and mechanical resilience. Such insights could markedly reduce recurrence rates and improve outcomes, especially for patients undergoing repeat abdominal surgeries.

C Can pharmacological or molecular interventions designed to rebalance collagen type I/III ratios or modulate matrix metalloproteinases (MMPs) and their inhibitors (TIMPs) improve postoperative scar integrity and reduce hernia formation?

Current therapeutic options for preventing or reducing IH formation primarily focus on mechanical solutions such as meshes or improved surgical techniques. However, collagen metabolism and MMP/TIMP regulation offer promising molecular targets for intervention. There is a notable lack of research exploring whether pharmacological agents or molecular therapies that specifically rebalance collagen type I/III ratios or stabilise the activity of MMPs could reduce hernia risk. Identifying and testing interventions aimed at molecular pathways involved in collagen remodelling could establish novel treatments or adjunct therapies to traditional mechanical surgical approaches, potentially leading to more robust scar formation and reduced recurrence rates. Examples include doxycycline, a broad‐spectrum MMP inhibitor shown to reduce pathological collagen degradation, or relaxin‐based therapies that modulate fibroblast phenotype and ECM remodelling in fibrotic conditions.

D How does aging and age‐related collagen remodelling impact the risk and characteristics of incisional hernias, and can age‐specific interventions mitigate this risk?

The incidence of IHs increases significantly with patient age, yet the precise relationship between aging, collagen metabolism and hernia formation remains inadequately explored. Aging affects collagen turnover, cross‐linking density and extracellular matrix organisation. Identifying how these age‐related changes specifically alter the risk, type and severity of IHs is critical. Understanding this relationship would facilitate the development of age‐specific preventive measures—whether mechanical (such as specialised surgical techniques and meshes tailored to older patients), pharmacological (targeting collagen stabilisation), or behavioural interventions (nutritional strategies, physical conditioning)—aimed explicitly at mitigating the elevated risk associated with aging.

E What are the exact molecular mechanisms by which mechanical signals translate into biochemical signals that regulate collagen deposition and maturation, specifically in abdominal fascia healing after surgery?

Though evidence strongly suggests that mechanical forces profoundly influence collagen deposition, orientation and maturation, the precise molecular mechanisms linking mechanical forces (mechanotransduction) to biochemical signals within abdominal fascia are incompletely understood. Detailed molecular studies are required to identify the key signalling pathways and mechanosensitive molecules (such as integrins, focal adhesion kinases, or growth factors) involved in this translation process. Clarifying these pathways could pave the way for innovative therapeutic approaches that artificially replicate beneficial mechanical signalling—potentially through controlled postoperative activity protocols or mechanotransduction‐targeted therapies—thereby promoting optimal collagen deposition and significantly reducing hernia incidence.

F How do differences in collagen metabolism and hernia risk vary by patient demographics such as gender, ethnicity, or underlying comorbidities (e.g., diabetes, obesity, or chronic inflammation)?

There is currently insufficient research exploring how demographic variables or common comorbidities affect collagen metabolism and consequently influence hernia susceptibility. Given the multifactorial nature of IHs, understanding these relationships at the molecular level is crucial. Investigating whether collagen type I/III ratios, cross‐linking processes, or MMP/TIMP dynamics vary systematically by gender, ethnicity, or comorbidity status could identify subpopulations at heightened risk. Such insights could lead to targeted preoperative risk assessments and more personalised approaches to surgery, postoperative care and preventive strategies, reducing both initial hernia formation and recurrence rates.

## 5 Mineralisation in Hernia Scar Tissue

Surgeons at the York Abdominal Wall Unit (YAWU) have observed white, discoloured, hard and brittle scar tissue around the hernia ring during IH repairs. The exact composition of this tissue has not yet been identified, but observations suggest mineralised connective tissue. Abnormal mineralisation is an under‐recognised yet potentially important contributor to scar failure and IH development. Calcification processes in scar collagen could predispose sutured layers to abnormal healing, ultimately permitting herniation. Despite these observations, no systematic studies have been conducted to investigate potential mineralisation in abdominal scars or its implications for hernia development. In this section, we examine two distinct but related processes—pathological calcification (PC) and heterotopic ossification (HO)—and discuss parallels with tendinopathy and ligament mineralisation.

### 5.1 Pathological Calcification (PC) and Dystrophic Calcification

Mineral deposits in both hard and soft tissues primarily consist of biological apatites, including phosphate and carbonate salts of calcium. These mineralisation processes can severely impact tissue mechanics. Gradual calcification of collagen fibrils, as described by Chatzipanagis et al. [59] reduces tensile capabilities, leading to tissue rigidity and brittleness. While this process is essential in hard tissues like bones and teeth, where collagen fibrils act as a template for mineral deposition, its occurrence in soft tissues often results in pathological conditions. These include disorganised, amorphous deposits known as PC or organised, bone‐like formations termed HO [60, 61]. Here, we briefly discuss both processes and reflect on them as potential contributing factors to incisional wound failure. PC has been implicated in various disorders, including atherosclerosis, calcific tendinitis, nephrolithiasis, several malignancies and arthritis [62, 63]. DC, the most common form of PC, occurs at sites of chronic inflammation, ischaemia, or previous damage. These conditions are relevant in primary and recurrent hernia cases. DC typically begins with damaged or dying cells acting as nucleation sites that attract dissolved minerals in the microenvironment, initiating mineral deposition in surrounding fibrous proteins like collagen. This process closely resembles the calcification of collagenous matrices during normal bone formation [60].

### 5.2 Mechanistic Overlaps With Tendinopathy and Ligament Calcification

**FIGURE 6:**
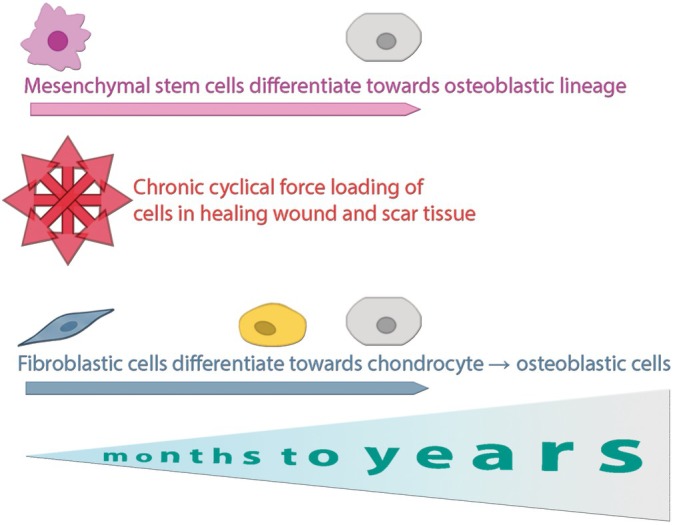
Proposed cellular differentiation pathways contributing to mineralisation in chronic abdominal wounds. Chronic cyclical mechanical loading and inflammatory signalling may promote fibroblast or mesenchymal stem cell differentiation towards osteoblast‐like phenotypes, leading to heterotopic ossification or dystrophic calcification over time.

Findings on DC in tendons and ligaments, tissues structurally similar to the linea alba, may provide clues to abdominal wound mineralisation, which may in turn be a component of the etiopathology of abdominal wall hernia. Microtrauma accumulation has been identified as a potential nucleation site for DC. For example, Tsukamoto et al. demonstrated that repetitive stretching in rat spinal ligaments induced ectopic cartilage and bony tissue development [64]. Human studies corroborate these findings, showing that cyclic stretching leads to fibroblast differentiation into chondrocytes and osteoblasts, coupled with increased expression of bone remodelling genes. Similar microcalcifications in spinal ligaments due to repetitive loading may mirror mineralisation in abdominal incisions subjected to cyclical abdominal pressure [65]. Mineralisation may be a slow, gradual process; Orzechowska et al. characterised spinal ligament calcification as crystal grains with a radius of up to 400 μm which developed in the first year and were impossible to detect by medical imaging. Larger particles (radius of 1000 μm) took up to 6 years to be detected by MRI or CT imaging [66]. Chronic tendinopathy is another example of repetitive micro‐strain below the failure threshold that induces chronic injury, calcification and eventual tendon rupture. The failure of the resident cells to repair the injured ECM can lead to advanced calcific tendonitis and tendon rupture. Tendon fibroblasts in such conditions often differentiate into a chondrocyte‐like phenotype that secretes mineral deposits. Notably, increased collagen type III and MMP activity, observed in calcific tendonitis, are also prevalent in several IH studies. These parallels suggest that abdominal scars in IH cases may follow similar calcification pathways. This process is hypothesised to occur gradually over months to years, driven by chronic cyclical loading of healing tissues and influenced by local inflammatory and biochemical signals. The schematic in Figure 6 illustrates proposed cellular differentiation pathways, from fibroblasts and mesenchymal stem cells towards chondrogenic or osteoblastic lineages, potentially contributing to tissue mineralisation in incisional scars.

### 5.3 Heterotopic Ossification (HO) in the Abdominal Wall

HO refers to the formation of bone fragments, complete with marrow elements, within soft tissues. Unlike PC, HO is a structured and progressive process often triggered by trauma or repetitive microtrauma. There is also evidence that DC may lead to HO and indeed these pathways are part of the same process. Kaplan et al. described this process as ‘osteogenic induction’, where multipotent mesenchymal stromal cells (MSCs) are recruited to healing tissues via newly formed blood vessels and differentiate into osteoblasts under local inflammatory cues. These have the ability to differentiate into osteoblasts under local inflammatory signals and calcify the fibrous connective tissue [67]. HO initially presents with swelling and pain; as the bony lesions mature the tissue becomes firm and can restrict motion [61]. Myositis ossificans is the most studied type of HO where bone forms in the muscle tissue, while ossification of the fascia (fasciitis ossificans) is a much rarer occurrence. Despite this, several case reports have been published discussing bony lesions in abdominal scars, and recent data suggests the incident rate after midline laparotomies can be as high as 26% [68]. HO is predominantly observed in vertical midline laparotomy scars, primarily in the upper abdominal region. Incidence rates as high as 26% following midline laparotomies have been reported. Lesions can range in size from millimetres to over 30 cm and often remain undetected until they cause discomfort or are incidentally identified during imaging [69, 70, 71]. Kim et al. highlighted that HO is frequently found in the linea alba, particularly in cases with a history of midline incisions [72]. While HO and PC share overlapping pathways, their roles in hernia formation remain unclear. Case reports, such as those by Lehrman et al. and Suleiman et al. [73, 74] describe IH cases with extensive HO surrounding the hernia ring. However, these remain isolated findings, and further, systematic microscopical studies are needed to clarify any causal relationships.

**Challenge:**

The microenvironment of incisional abdominal wounds can potentially lead to uncontrolled mineral deposition impacting the structure and physical strength of the forming tissue. To identify a possible role of this mineralisation process following questions need to be addressed:

- What is the precise biochemical and structural composition of the mineralised tissue observed in incisional hernia scars, and how does this composition compare to other known types of pathological calcification (PC) or heterotopic ossification (HO)?

Despite surgeons' repeated observations of white, hardened, mineral‐like scar tissue during hernia repairs, the exact biochemical and structural composition of this tissue remains uncharacterised. Understanding the exact mineral composition—whether primarily apatite‐based calcifications similar to those found in vascular calcifications, DCs, or bone‐like structures indicative of HO—is essential. This detailed compositional analysis could clarify the biological origin of these minerals, help differentiate between PC and HO processes, and provide insights into their possible roles in hernia pathogenesis. Clarifying these distinctions is important, as different mineralisation pathways may require distinct prevention and treatment approaches.

B What molecular and cellular signals trigger mineral deposition in abdominal wall scars following surgical incision, and how do these signals differ between patients who develop calcification and those who do not?

Mineralisation in soft tissues is generally regulated tightly to prevent inappropriate calcification. Therefore, understanding what triggers the pathological deposition of minerals within the collagen‐rich matrix of abdominal wall scars is crucial. Investigating specific cellular and molecular pathways involved—such as osteogenic signalling molecules (BMPs, Runx2), pro‐inflammatory cytokines (TNF‐α, IL‐6), or extracellular calcium/phosphate imbalances—could reveal why mineralisation occurs in some patients but not others. This knowledge could identify predictive biomarkers and lead to targeted therapies capable of preventing or reversing calcification, thereby reducing the risk of abnormal tissue rigidity and subsequent hernia development.

C How does repetitive mechanical stress from daily activities (such as coughing, bending, or lifting) contribute to initiating or accelerating mineralisation processes in abdominal wound scars, compared with similar mechanical influences documented in tendons and ligaments?

Evidence from tendinopathies and spinal ligament calcification suggests that repetitive mechanical stress may induce mineralisation through chronic microtrauma. However, it remains uncertain whether similar mechanisms underlie abdominal scar mineralisation after surgery. Elucidating whether repetitive intra‐abdominal pressure changes, commonplace after laparotomy, directly stimulate mineralisation through mechanotransduction pathways in abdominal fascia is critical. Establishing this link could help identify at‐risk patients based on their activity profiles or surgical history and inform postoperative care recommendations aimed at mitigating repetitive micro‐injury, potentially reducing calcification risk and subsequent hernia development.

D Is mineralisation in incisional hernia scar tissue primarily a localised phenomenon restricted to the site of surgery, or does it reflect broader systemic disturbances in mineral homeostasis and connective tissue metabolism?

The presence of mineralisation in scar tissue might be a local phenomenon triggered by trauma and inflammation, but it could also signify systemic imbalances in mineral or connective tissue metabolism. Determining whether calcification is limited to surgically disrupted areas or also occurs in distant, unaffected tissues could offer important clues. Identifying systemic markers or disturbances could lead to broader screening methods, allowing clinicians to detect mineralisation‐prone patients earlier and implement systemic preventative strategies, thus reducing the risk of recurrent or multifocal calcification complications.

E Can imaging or diagnostic tools effectively detect early‐stage mineralisation within abdominal scars before they become clinically significant, and if so, how can this information influence clinical management and prevent hernia recurrence?

Current medical imaging techniques, such as ultrasound, CT or MRI, often fail to detect early, microscopic mineral deposits that may contribute to later hernia formation. However, advances in imaging modalities (such as Raman spectroscopy, high‐resolution ultrasound, or advanced CT/MRI techniques) could potentially identify mineralisation at early stages. Determining the efficacy of such non‐invasive diagnostic tools to detect subtle mineralisation changes before clinical manifestation could transform postoperative monitoring. This capability would facilitate earlier intervention, personalised postoperative strategies and potentially pharmaceutical or mechanical prophylactic interventions, thereby substantially reducing hernia incidence and improving patient outcomes.

F Can therapeutic interventions targeting calcification pathways (e.g., bisphosphonates, anti‐inflammatory therapies, or novel molecular agents) prevent or reverse mineral deposition in abdominal scars, thus improving tissue mechanics and decreasing hernia risk?

Currently, there are no validated therapeutic strategies specifically targeting mineralisation processes in abdominal scars. Considering the significant mechanical implications of calcified scar tissue for hernia susceptibility, there is an urgent need to investigate potential therapeutic agents. Evaluating drugs known to influence calcification processes in other diseases, such as bisphosphonates, anti‐inflammatory agents, or novel molecular inhibitors of mineral deposition, could uncover promising strategies for reducing or preventing abnormal mineralisation in abdominal scars. Developing these therapeutic approaches could significantly improve postoperative outcomes by preserving tissue elasticity, preventing scar brittleness and ultimately reducing hernia occurrence.

## 6 Future Research

Throughout this review, several key themes emerged regarding key challenges regarding the pathogenesis of IH. These gaps provide exciting opportunities for interdisciplinary research involving surgeons, physicists and materials scientists, employing advanced analytical techniques to deepen our understanding and improve clinical outcomes. Although diverse in nature, these challenges can be grouped into thematic clusters.

*Theme 1: Local* Versus *Systemic Factors—*A fundamental uncertainty remains whether collagen and mineralisation abnormalities in hernia formation reflect predominantly local responses to surgical trauma or broader systemic disturbances. Clarifying this through detailed molecular profiling (e.g., genetic sequencing, transcriptomics) and advanced biochemical imaging techniques could enable targeted interventions and risk stratification.

*Theme 2: Molecular and Cellular Mechanisms of Collagen Remodelling—*The exact cellular signals that regulate collagen type I/III deposition and maturation remain unclear, particularly in abdominal wound healing. Employing single‐cell RNA sequencing and targeted gene editing could help identify these pathways. Advanced microscopy methods, such as electron microscopy, could characterise collagen fibril morphology and cross‐linking at nanoscale resolution, providing critical mechanistic insights.

*Theme 3: Mineralisation in Hernia Scar Tissue—*The composition and triggers of mineral deposition observed clinically in hernia scars are poorly understood. Investigating these deposits through Raman spectroscopy would precisely characterise their biochemical nature. Additionally, electron microscopy could reveal structural properties and interactions with collagen fibrils, clarifying their mechanical impact.

*Theme 4: Mechanical Influences on Tissue Healing—*How mechanical forces translate into biochemical signals influencing collagen deposition, maturation and mineralisation is an unresolved issue. Biomechanical modelling and Raman spectroscopy could track collagen alignment and maturation under varying mechanical stresses, identifying mechanical conditions for optimal scar formation. This may also inform postoperative rehabilitation following CAWR.

*Theme 5: Early Detection and Diagnostic Innovations—*A significant challenge is identifying early‐stage abnormal collagen remodelling or mineralisation in abdominal scars. Non‐invasive Raman spectroscopy provides real‐time biochemical analysis of healing tissue, potentially identifying incipient mineralisation or abnormal collagen changes before clinical symptoms arise. Also, postoperative ultrasound may be a valuable tool in identifying developing fascial defects or dynamic abdominal wall strain patterns before hernia manifestation. Its non‐invasive and relatively low‐cost nature allows for serial assessments, particularly in high‐risk patients.

*Theme 6: Therapeutic and Biomaterial Advancements—*Finally, novel therapeutic interventions and biomaterials are needed to address collagen and mineralisation abnormalities directly. Multidisciplinary collaboration employing advanced material characterisation techniques (e.g., Raman spectroscopy, electron microscopy, biomechanical testing) could guide the development of biomimetic meshes and targeted pharmacological therapies, specifically designed to improve scar mechanical properties and reduce hernia recurrence.

Understanding the biomechanical and molecular contributors to IH formation has tangible implications for surgical practice. At‐risk populations include patients with obesity, chronic respiratory disease (e.g., COPD), connective tissue disorders (e.g., Ehlers–Danlos syndrome), diabetes and those undergoing emergency or contaminated procedures. Interventions that may mitigate IH risk include the use of slowly absorbable or non‐absorbable sutures, small‐bites suture techniques, prophylactic mesh in high‐risk patients and targeted prehabilitation to optimise modifiable risk factors. Future therapies may focus on modulating collagen metabolism (e.g., doxycycline as an MMP inhibitor) or delivering mechanical cues to enhance scar strength. These findings may support risk‐stratified repair protocols and justify future trials of adjunctive therapies informed by patient‐specific biomechanical risk profiles. Recent years have also seen increased interest in biologic materials derived from non‐human or human tissue sources. Examples include acellular fish skin matrices, bovine pericardium, human dermis and placental membranes, which are processed to retain structural collagen while removing immunogenic components. These biologic ECMs are typically deployed in onlay or underlay configurations and are designed to act as scaffolds for host tissue ingrowth, angiogenesis and remodelling. They are particularly favoured in contaminated or high‐risk fields where synthetic mesh use is cautioned against and may reduce chronic inflammation and fibrosis. While it is beyond the scope of this review to comment in detail on the performance or indications of these emerging materials, we acknowledge the important role they may play in future hernia repair strategies, particularly in biologically hostile or contaminated fields. Their integration within a regenerative repair paradigm aligns with the broader shift from reinforcement to restoration in abdominal wall surgery.

## 7 Conclusion

IH remain a common and complex complication of abdominal surgery, underpinned by an interplay of mechanical disruption, altered wound healing, collagen dysregulation and, potentially, pathological tissue mineralisation. While previous studies have shed light on some of these processes, much remains unclear. In this narrative review, we have re‐examined these phenomena through the lens of our interdisciplinary team, drawing on surgical experience, materials science and physics. By combining clinical observations with insights into collagen ultrastructure, biomechanical signalling and mineral deposition, we have identified unanswered questions that demand further exploration. These range from the molecular determinants of collagen remodelling to the triggers and consequences of tissue mineralisation, to the challenge of replicating the anisotropic properties of native abdominal tissues in surgical repair. Advancing our understanding in these areas will require new investigative approaches. Techniques such as Raman spectroscopy and electron microscopy offer powerful, underutilised tools for characterising collagen architecture, identifying early mineralisation and probing tissue mechanics at a micro‐ and nanoscale. Ultimately, the path forward lies in embracing a more integrated, cross‐disciplinary model of investigation. This will be one that combines surgical insight with precision imaging and materials analysis. Mechanistic insights from this review may help define high‐risk subgroups such as individuals with obesity, advanced age, smoking history, history of wound infection, or comorbid diabetes mellitus or connective tissue disorders (e.g., Ehlers–Danlos syndrome). Improved understanding of wound healing and matrix remodelling may refine surgical decision‐making, informing optimal closure techniques, guiding mesh material selection based on biomechanical compatibility and facilitating patient‐specific rehabilitation strategies to mitigate hernia recurrence. For example, rehabilitation protocols could be adapted to support tension‐modulated collagen alignment, and early identification of abnormal healing trajectories could enable personalised intervention before failure occurs.

## Conflicts of Interest

The authors declare no conflicts of interest.

## Supporting information


**Supporting File S1:** Supporting Information.

## Data Availability

Data sharing not applicable to this article as no datasets were generated or analysed during the current study.
