# Supplementary material for: Incisional Hernia Development: Wound Healing Gone Wrong?
Source: Wound Repair Regen. 2026 Mar 5;34(2):e70131. doi: 10.1111/wrr.70131 (PMC12963529; doi:10.1111/wrr.70131)
Supplement: Supplementary file 1 — Supporting File S1: Supporting Information. [file WRR-34-0-s001.docx]

**Supplementary file 1 - Methods**

**Methods**

This paper presents a narrative literature review undertaken by a multidisciplinary team of surgeons, a physicist, and a materials science expert. Our goal was to critically explore and interpret the underlying mechanisms of IH development, with particular focus on wound healing, collagen remodelling, and tissue mineralization. Rather than aiming to systematically catalogue all available studies, this review synthesizes relevant findings from the literature in the context of our clinical experience and scientific perspectives.

To guide the review, we performed a targeted search of the biomedical literature using Ovid MEDLINE (1946 to January 2025) and Embase (1974 to January 2025). The search strategy incorporated both controlled vocabulary terms (e.g., MeSH, Emtree) and free-text keywords to capture literature addressing the aetiology, pathophysiology, and mechanical failure of abdominal wall healing following laparotomy. The search strategy included combinations of terms such as "*incisional hernia*", "*pathophysiology*", "*collagen*", "*wound healing*", and "*mineralization*". Retrieved records were imported into reference management software (Covidence), where duplicates were removed. Articles were then screened for relevance based on their contribution to one or more of the key conceptual themes. This included histological, biochemical, and biomechanical studies in both human and animal models, as well as publications exploring connective tissue disorders, hernia recurrence, and surgical repair techniques. We drew particular attention to publications offering insight into fascia remodeling, collagen subtypes and cross-linking, aberrant healing responses, and the potential for pathological mineralization. Unlike systematic reviews, we did not apply rigid inclusion or exclusion criteria. Rather, articles were selected based on their relevance, depth of insight, and contribution to emerging hypotheses. Where appropriate, we highlight both well-established findings and unresolved questions. Each section concludes by identifying conceptual gaps that merit further investigation.

Our overarching aim is to offer a structured, cross-disciplinary interpretation of IH pathogenesis. In doing so, we hope to lay the groundwork for future research. As the key function of the abdominal wall (AW) is the containment of the intestines while allowing for adaptation to mechanical forces it needs to provide mechanical stability and flexibility enabled by an intricate combination of different layers. Hence, we need to understand the multilayered structure of the abdomen and the specific role of the AW.
